# Homology-based reconstruction of regulatory networks for bacterial and archaeal genomes

**DOI:** 10.3389/fmicb.2022.923105

**Published:** 2022-07-19

**Authors:** Luis Romero, Sebastian Contreras-Riquelme, Manuel Lira, Alberto J. M. Martin, Ernesto Perez-Rueda

**Affiliations:** ^1^Licenciatura en Ciencias Genomicas, Universidad Nacional Autonoma de Mexico, Cuernavaca, Mexico; ^2^Laboratorio de Biología de Redes, Centro de Genómica y Bioinformática, Facultad Ciencias, Ingeniería y Tecnología, Universidad Mayor, Santiago, Chile; ^3^Cómputo Académico, Facultad de Ciencias - UMDI-Sisal, Sede Parque Científico y Tecnológico de Yucatán, Universidad Nacional Autónoma de México, Mérida, Mexico; ^4^Instituto de Investigaciones en Matemáticas Aplicadas y en Sistemas, Universidad Nacional Autónoma de México, Unidad Académica Yucatán, Mérida, Mexico

**Keywords:** regulatory networks, orthology, transcription units, regulatory modules, genomics

## Abstract

Gene regulation is a key process for all microorganisms, as it allows them to adapt to different environmental stimuli. However, despite the relevance of gene expression control, for only a handful of organisms is there related information about genome regulation. In this work, we inferred the gene regulatory networks (GRNs) of bacterial and archaeal genomes by comparisons with six organisms with well-known regulatory interactions. The references we used are: *Escherichia coli* K-12 MG1655, *Bacillus subtilis* 168, *Mycobacterium tuberculosis*, *Pseudomonas aeruginosa* PAO1, *Salmonella enterica* subsp. *enterica* serovar *typhimurium* LT2, and *Staphylococcus aureus* N315. To this end, the inferences were achieved in two steps. First, the six model organisms were contrasted in an all-*vs*-all comparison of known interactions based on Transcription Factor (TF)-Target Gene (TG) orthology relationships and Transcription Unit (TU) assignments. In the second step, we used a guilt-by-association approach to infer the GRNs for 12,230 bacterial and 649 archaeal genomes based on TF-TG orthology relationships of the six bacterial models determined in the first step. Finally, we discuss examples to show the most relevant results obtained from these inferences. A web server with all the predicted GRNs is available at https://regulatorynetworks.unam.mx/ or http://132.247.46.6/.

## Introduction

Bacterial and archaeal organisms respond to diverse stimuli *via* the subtle mechanism of regulation of gene expression at the transcriptional level, and this involves DNA-binding proteins known as transcription factors (TFs). These proteins act by interacting with specific sites, usually upstream of the transcription start site, inducing or blocking access of the RNA polymerase to the promoter. In general, when a TF binds at a site that overlaps the promoter region of a gene, the system is repressed; when the binding site is upstream of the promoter, the system is activated (Browning and Busby, 2016). In addition, this regulatory system is coordinated with the sensing of endogenous or exogenous stimuli by these regulatory proteins, i.e., they have the ability to sense diverse conditions for the cell to contend against environmental changes. For instance, in the bacterium *Escherichia coli* K-12, approximately three-quarters of TFs respond directly to extracellular signals through phosphorylation and binding to small molecules, such as allolactose or maltose (Balderas-Martínez et al., 2013).

In this context, the regulatory system can be conceptualized as a circuit, where one TF can regulate multiple Target Genes (TGs) and multiple genes can be regulated by one or diverse TFs, all of them assembled into a gene regulatory network (GRN). In GRNs, nodes represent genes and the connections between them indicate that the TF-encoding gene regulates another gene; this type of network can be represented by directed graphs (Karlebach and Shamir, 2008).

To date, GRNs have been determined for only a few bacterial models from three different phyla: Proteobacteria, including *Escherichia coli* K-12, *Salmonella enterica* subsp*. enterica* serovar *typhimurium* LT2, and *Pseudomonas aeruginosa* PAO1; Firmicutes, including *Bacillus subtilis* 168 and *Staphylococcus aureus* N315; and Actinobacteria, including *Mycobacterium tuberculosis*. The lack of GRNs for most microorganisms is due to the fact that reconstruction depends largely on experimental data. Therefore, the inference or expansion of regulatory relationships between TFs and their TGs in organisms beyond the bacterial models will allow us to understand diverse biological processes, such as cell growth, response to environmental changes, or cell division, among others.

In this regard, various approaches have been explored to reconstruct regulatory networks in bacteria, such as RegPrecise (Novichkov et al., 2010), with a large amount of information available for regulons of diverse organisms, or the work of Castro-Melchor et al. (2010) based on the transcript and functional similarities to infer regulatory networks in *Streptomyces coelicolor*, among others. However, the main limitations of these reconstructions are associated with the experimental information data.

Hence, to determine the GRNs in bacterial and archaeal genomes with no information on their regulatory interactions, we mapped orthologous interactions among the six bacterial models to identify novel TF-TG interactions. Next, we used a guilt-by-association approach to infer the GRNs for 12,230 bacterial and 649 archaeal genomes, based on TF-TG orthology relationships of six bacterial species with well-known regulatory interactions and Transcription Unit (TU) assignments (i.e., operonic organization). The “guilt-by-association” principle has been applied to deduce functional relationships (Oliver, 2000), and used to predict gene function in various types of biological networks, for example in virulence factors of the bacterial pathogen, *Aeromonas veronii* (Li et al., 2021). The reconstructed networks were evaluated in terms of their topological properties, identifying TFs as hubs, modules, and co-regulated genes. Thus, our approach allowed us to confer a degree of accuracy regarding the existence of each inferred interaction. Therefore, the predicted interactions must be considered as a starting point to further exploration, both *in silico* and experimentally. We suggest that posterior analysis must consider the identification of DNA-binding sites upstream the probable regulated gene or a functional analysis with Gene Ontology and global expression profiles, as it has been already suggested in other cellular systems beyond bacteria and archaea (Chen, 2017). Finally, a web server with all the predicted GRNs is available to the scientific community at https://regulatorynetworks.unam.mx/ or http://132.247.46.6/.

## Data and methodology

### Genomes used for reference

The information for six bacterial genomes used in this work was downloaded from either the NCBI server or RegulonDB: *E. coli* K-12 MG1655 (NC_000913.3, GCF_000005845.2), *B. subtilis* 168 (GCF_000009045.1), *P. aeruginosa* PAO1 (GCF_000006765.1), *S. typhimurium* LT2 (GCF_000006945.2), *S. aureus* N315 (GCF_000009645.1), and *M. tuberculosis* (GCF_000195955.2). For each genome, the FASTA sequence was obtained from the “gbff/gbk” files parsed with an *ad hoc* program (Supplementary material, ParserGBK.py), to add the appropriate label in the header: NCBI gene ID, local gene ID, gene name, product description, and organism name. Sequences with missing information were annotated as “NODATA.” In addition, the 12,230 genomes of bacteria and 649 archaeal genomes were downloaded from the NCBI RefSeq genome database on May 18, 2021, to infer their GRNs.

### Gene regulatory interactions

The regulatory interactions were obtained from specialized databases [DBTBS for *B. subtilis* release 5 (Sierro et al., 2008),^1^ RegulonDB release 10.9 for *E. coli* (Santos-Zavaleta et al., 2019a),^2^ *M. tuberculosis* (Kapopoulou et al., 2011; Sanz et al., 2011), RegulomePA release 1.0 for *P. aeruginosa*,^3^ Salmonet release 2.0 for *S. typhimurium* LT2 (Métris et al., 2017), and for *S. aureus* N315 (Ravcheev et al., 2011; Poudel et al., 2020)] and posteriorly homogenized, following the same format: First column corresponds to the assigned number by regulatory interaction per organism; second column, TF associated; third column, Target gene; and the other columns indicate the annotations derived from the original networks (Supplementary material). These GRNs are summarized in Table 1.

**Table 1:** Total new interactions per organism.

Contribution source →	*B. subtilis 168*	*E. coli K-12*	*P. aeruginosa PA01*	*S. typhimurium LT2*	*S. aureus N315*	*M. tuberculosis H37Rv*	TUs	New interactions
Network contributed ↓								
*B. subtilis 168* (2738)	–	395 (21.69%)	34 (1.86%)	255 (14.00%)	206 (11.31%)	286 (15.70%)	828 (45.46%)	1821
*E. coli K-12* (3616)	248 (14.79%)	–	157 (9.36%)	600 (35.79%)	125 (7.45%)	193 (11.51%)	393 (23.62%)	1,676
*P. aeruginosa PA01* (998)	139 (5.56%)	1,117 (44.69%)	–	709 (28.37%)	92 (3.68%)	331 (13.24%)	679 (27.17%)	2,499
*S. typhimurium LT2* (2969)	259 (10.71%)	1,135 (46.95%)	140 (5.79%)	–	124 (5.13%)	238 (9.84%)	608 (25.15%)	2,417
*S. aureus N315* (709)	355 (43.88%)	173 (21.38%)	8 (0.98%)	109 (13.47%)	–	79 (9.76%)	177 (21.87%)	809
*M. tuberculosis H37Rv* (2637)	70 (9.02%)	242 (31.18%)	17 (2.19%)	140 (18.04%)	22 (2.83%)	–	405 (52.19%)	776

The number of interactions, the contribution percentage of each organism (row “contribution”) to the new interactions, and the extension by TU assignment, is indicated. The number of interactions in the original network is indicated in brackets (first column).

### Ortholog identification

The protein sequences from each model organism were used as reference to identify the orthologs in an all-*vs*-all genomes fashion using the program Proteinortho (Lechner et al., 2011) with the following parameters: E-value ≤10^5^, coverage ≥70%, and identity of ≥25%, as previously described for the identification of TFs (Flores-Bautista et al., 2020).

### Transcription units

The predictions of Transcription Units (TUs) or operons were obtained using the method described by Moreno-Hagelsieb and Collado-Vides (2002). In brief, the predictions were based on the transcription direction and the intergenic distance (shorter intergenic distances and in the same direction for genes in the same TU).

### Inference of GRNs

**Figure 1:**
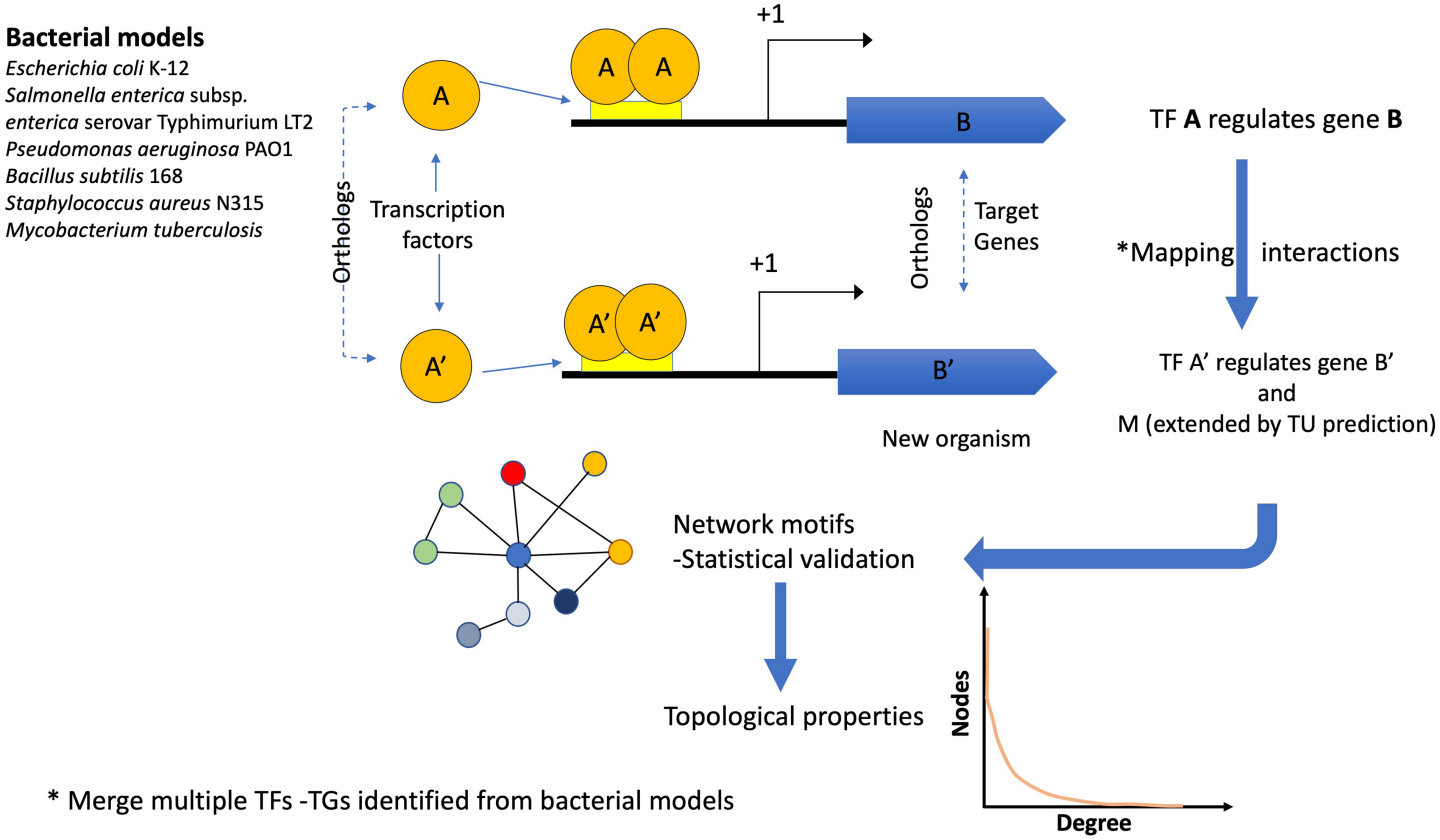
Flux diagram showing the inference of the GRNs. Six bacterial models were used to infer the GRNs in 12,230 bacterial and 649 archaeal genomes. If the pair A (TF) – B (TG) in a reference genome is identified (by orthology) in a new genome A′ (TF) –B′ (TG), the interaction is assigned. In addition, if the TG identified in the new genome is the first one in the TU, the interaction is extended to the other gene(s). One interaction in a new genome can be derived from one or more bacterial models. Finally, the reconstructed networks were evaluated in terms of their topological properties.

The reference genomes were used to scan the 12,230 bacterial and 649 archaeal genomes to identify their orthologs and map their interactions considering the following criteria: If the orthologs of the TF and its TG of the model organism were found in a new genome, the interaction was assigned using guilt by association. In a second step, predicted TUs were used to expand the TF-TG interactions as follows: If the first gene of the orthologous TG in an organism corresponded to the first gene in the TU, the other genes belonging to the TU were associated with the same TF. Finally, each network was integrated using all the ortholog assignments with the six reference GRNs. All the network interactions can be inferred by running the script *pipeline.sh*, provided as Supplementary material and Figure 1.

### Regulatory modules

The GRNs were analyzed by using Cytoscape (Shannon et al., 2003; Otasek et al., 2019) to obtain their degree, clustering coefficient, and other centrality metrics. Hubs were obtained by using networkX from python (Hagberg et al., 2008). In addition, to identify transcriptional co-regulators and modules in a GRN, the CoReg software was used. In brief, CoReg calculates gene similarities based on the number of common neighbors of any two genes in the network (Song et al., 2017).

### Web server

The GRNs inferred for all the bacterial and archaeal genomes are available through the web server at https://regulatorynetworks.unam.mx/, which is built on HTML5, JQuery, and Php languages, while the data are stored in a MySQL database. For the data display, we use the Cytoscape JS (Franz et al., 2016) framework due its capabilities to represent nodes and edges of the network with determined properties, allowing users to change forms, colors, and layer visualization of the network.

### Method performance and statistical analysis

GRNs were compared using two different approaches to establish the reliability of the approach, one based on the ability to recover edges and the second focusing on the ability to recover network motifs (Milo et al., 2002) by comparing the six reference networks with networks for the same genomes generated using our approach.

First, based on the orthologous annotations made with Proteinortho, we created a GRN for each of the reference bacteria using a naming convention that ensures that genes that are orthologous among them share the same name in each of the six GRNs used as reference. Then, we created networks for each reference organism based on the regulations transferred from the other five GRNs, again using the consensus gene names. GRNs with consensus gene names were then compared, following two procedures implemented in LoTo (Martin et al., 2017). We employed binary classification metrics to evaluate the similarities between pairs of GRNs as follows: Edges present in both compared networks are considered true positives (TPs), genes only present in one of the networks are false negatives (FNs) if they are only in the reference network, True Negatives (TNs) are the edges absent in both compared networks, and false positives (FPs) if they appear only in the network we compared with the reference. This edge-based approach is used to compare predicted GRNs versus reference networks, and it indicates overall network similarity (The DREAM5 Consortium et al., 2012). The second approach relies on the presence or absence of the motifs defined by Milo et al. (2002) that have been related to functional patterns in GRNs. Instead of considering TF-gene interactions, in this second approach, we considered TP motifs present in both compared networks, FN motifs are only found in the reference network, and FPs are only present in the network compared against the reference GRN.

LoTo calculates several metrics, but here we only focused on the most employed ones:


Precision(P)=TP/(TP+FP)

$$\mathrm{Recall}\;\left(R\right)=\mathrm{TP}/\left(\mathrm{TP}+\mathrm{FN}\right)$$

and


$$\mathrm{F1}=\mathrm{2PR}/\left(P+R\right)$$

To establish a baseline and determine whether the results from our approach are significant versus what can be expected by chance, we also created a protocol to determine the expectancy of a transferred TF-gene regulation by chance. We randomized the names TFs for the whole inferred networks 10,000 times to calculate expected TP, FP, TN, and FN values by comparing these randomized networks against their reference counterparts. This protocol ensures comparisons of random networks with the same characteristics, e.g., edges, TFs, and genes, against their actual reference. We then employed a G-test as implemented in SciPy (Virtanen et al., 2020) to determine whether the observed number of edges considered TP, FP, TN, and FN can be from the same distribution as that observed for the predicted networks without randomization.

## Results and discussion

### Identification of new interactions in bacterial models

In order to evaluate and expand the GRNs of the six model organisms, the number of TFs, TGs, and their interactions was determined. To do this, we downloaded six GRNs, and their interactions were displayed by using Cytoscape. In this work, we considered TFs as those proteins that activate or repress gene expression but do not belong to the transcriptional basal machinery; therefore, sigma factors, antiterminators, terminators, and sensor proteins, among other proteins, were excluded from the resulting data set (Martínez-Núñez et al., 2013). Table 2 shows the number of interactions associated with each organism. The most studied bacterial species, *E. coli* K-12, has 3,616 interactions based on experimental evidence, followed by *S. typhimurium* LT2 (2,969 interactions) and *B. subtilis* with 2738TF-TG interactions, whereas the GRN of *S. aureus* contains the smallest number of interactions, with 709. This difference could be a consequence of the experimental evidence accumulated over the years and the number of experiments carried out and performed with each organism; i.e., there is a bias inherent to the experimental analysis towards specific organisms. For instance, in a recent collection of 668 experimentally characterized TFs in bacteria and archaea organisms (Flores-Bautista et al., 2020), 33.5% was associated with *E. coli K12,* 23% with different strains of *M. tuberculosis*, and 19% with *B. subtilis* 168; i.e., 76% of the complete collection is concentrated in few organisms; in contrast, 24% of the collection is distributed among 78 different prokaryotes. This contrast in the information is also evident in more general databases, such as UniProtKB/Swiss-Prot, where *E. coli K-12* is the bacterial organism with more proteins deposited and curated manually in the database.^4^

**Table 2:** Single edge comparisons between the six reference networks employed in this work and their counterparts generated following our homology-based transfer approach from the other remaining networks.

Organism	TP	FP	FN	*R*	*P*	*F*1	*p*-value
*B. subtilis 168*	254	499	2,447	0.094	0.3373	0.147	4.01e–258
*E. coli K-12*	1,538	709	1,971	0.4383	0.6845	0.5344	0.0
*P. aeruginosa PA01*	51	202	938	0.0516	0.2016	0.0822	9.46e–39
*S. typhimurium LT2*	1,491	666	1,394	0.5168	0.6912	0.5914	0.0
*S. aureus N315*	229	237	466	0.3295	0.4914	0.3945	9.45e–229
*M. tuberculosis H37Rv*	71	138	2,494	0.0277	0.3397	0.0512	5.99e–52

Precision (P), Recall (R), and F1 were calculated using the true positive (TP), false positive (FP), and false negative edges (FN). P-value of the G-test indicates the significance of the differences between the averaged counts of TP, FP, TN, and FN in the 10,000 randomizations of the inferred networks and the results shown in the table.

To determine the number of interactions shared between the six model organisms, we first used the program Proteinortho to assign orthology relationships between all proteins in the proteome of each bacterium. Once orthologous proteins were determined, we inferred regulatory interactions between organisms based on the presence of an orthologous TF and an orthologous target of that TF in the model GRN. In the second step, the interactions were expanded by using the TU assignments, as described in Materials and Methods. This comparison showed that *E. coli* and *S. typhimurium* LT2 share a high number of interactions, because of their phylogenetic closeness. In contrast, the actinobacterium *M. tuberculosis* is the organism with the lowest number of shared interactions with the other bacterial models as a consequence of its phylogenetic distance; only 12% (in average) of its interactions are shared with other bacteria (see Supplementary material).

In order to infer new interactions among the six bacterial genomes, they were compared and their interactions were assigned based on the presence of the TF-TG orthologous pairs. In this regard, Table 1 shows the number of new assignments and their proportion per organism. From this analysis, we found between 776 and 2,499 new interactions, with *S. typhimurium* LT2 and *P. aeruginosa* the organisms determined to have more new interactions inferred. These larger numbers for *S. typhimurium* LT2 and *P. aeruginosa* are probably a consequence of their phylogenetic closeness with *E. coli* K-12 (Fukushima et al., 2002) in comparison to the other organisms used as models. It is important that some regulatory interactions were found in more than one organism; therefore, the sum of the rows may not correspond to the total number of new interactions, as is the case for the regulator PhoB (NP_414933.1) of *E. coli* K-12, which regulates the cytochrome bd-I ubiquinol oxidase subunit (NP_415262.1), as inferred from the interactions previously described in the *B. subtilis* and *M. tuberculosis* networks.

### Performance estimation of the approach

Regarding the reliability of interactions predicted by our approach, we compared networks with only TF-TG interactions derived from homology relationships for each of the six species with the respective reference GRNs. The comparisons were made by considering this to be a binary classification problem, and thus, edges (and graphlets) in both the reference network and the predicted GRN are TPs, edges only in the reference are FNs, and edges only in the predicted network are FPs. These results, shown in Table 2 for single edges and in Table 3 for graphlets, indicate a varying range of values depending on the compared bacteria. For recall (R), the rate of recovered TF-TG interactions ranged from 0.028 for *M. tuberculosis* to 0.52 for *S. enterica*, whereas precision (P), which indicates the likelihood that the existence of an edge is correctly predicted, ranged from 0.20 for *P. aeruginosa* to 0.69 for *S. enterica*. These results are significantly different from those expected by chance, as shown by the very low *p*-values obtained with the G-test. When the same metrics for the presence and absence of graphlets were used (Table 3), we found a similar trend for each model GRN but with lower values for each metric. Lower values for the metrics calculated with graphlets are expected, since a single edge that differs between two networks often affects various graphlets.

**Table 3:** Graphlets absence comparison between the six reference networks employed in this work and their counterparts generated following our homology-based transfer approach from the other remaining networks.

Organism	TP	FP	TN	FN	*R*	*P*	*F*1
*B. subtilis 168*	2,241	10,008	622,878,341	145,210	0.0152	0.183	0.0281
*E. coli K-12*	57,366	38,545	619,477,397	185,907	0.2358	0.5981	0.3383
*P. aeruginosa PA01*	101	2,815	73,261,825	12,989	0.0077	0.0346	0.0126
*S. typhimurium LT2*	56,206	50,622	362,053,161	134,678	0.2945	0.5261	0.3776
*S. aureus N315*	2087	5,176	17,864,376	19,578	0.0963	0.2873	0.1443
*M. tuberculosis H37Rv*	215	1876	117,513,083	234,607	0.0009	0.1028	0.0018

### The expanded GRNs identified new TF-TG interactions

Based on the expanded networks, we identified new TF-TG interactions described in Table 4 that must be exhaustively analyzed. In this regard, we found an increase in the number of targets, TFs, nodes, and interactions for all the bacterial and archaeal extended networks. For instance, for *M. tuberculosis* H37Rv, there was an increase of 776 new interactions (305 new TGs and 31 new TFs), whereas for *B. subtilis*, 1821 new interactions (36 new TFs and 553 new TGs) were identified. Therefore, we performed a literature search to find evidence to support our predictions. Based on these searches, and considering the 1,676 new interactions for *E. coli* K-12 (56 new TFs and 570 new TGs), we identified that 179 of these interactions have been described in the literature (Supplementary material); however, they are not deposited in RegulonDB. In particular, we found that the interaction of SoxS and *ompW* in the GRN of *E. coli* and inferred from *S. enterica* has been experimentally described. In *E. coli*, *ompW* is regulated by three TFs, as described in RegulonDB; however, we found that it could be also regulated by SoxS (Zhang et al., 2020) in a negative fashion.

**Table 4:** Comparisons between experimentally and inferred GRNs.

Organism	Target counts	Target counts extended _tu	TF counts	TF counts extended_tu	Node count	Node count extended _tu	Edge count	Edge count extended _tu
*B. subtilis 168*	1748	2,301	191	227	1799	2,339	2,738	4,559
*E. coli K-12*	1,618	2,188	196	252	1,670	2,224	3,616	5,292
*P. aeruginosa PA01*	604	1701	124	236	638	1741	998	3,497
*S. typhimurium LT2*	1,640	2,371	131	224	1,670	2,404	2,969	5,386
*S. aureus N315*	584	973	51	101	598	990	709	1,518
*M. tuberculosis H37Rv*	1,405	1710	76	107	1,431	1733	2,637	3,413

Columns as are follows: Genome name; columns 1, 3, 5, and 7 indicate the Targets, TFs, nodes, and number of interactions identified in the original networks; columns 2, 4, 6, and 8 indicate the Targets, TFs, nodes, and number of interactions identified in the extended networks.

We also found a new interaction, where CpxR could be regulating *tar* gene. This TF together CsgD has been described in bacterial adhesion, and belongs to the stationary-phase response (Santos-Zavaleta et al., 2019b). Experimental evidence suggests that CpxR and CsgD repress the transcription of *fliA*, *flgM*, and *tar* (Dudin et al., 2014), in addition to *bglg* and *bglb* (Mattéotti et al., 2011), and PdhR and *lipA* (Kaleta et al., 2010). These regulatory interactions identified by our orthologs inferences have not been documented in RegulonDB.

### Web server

**Figure 2:**
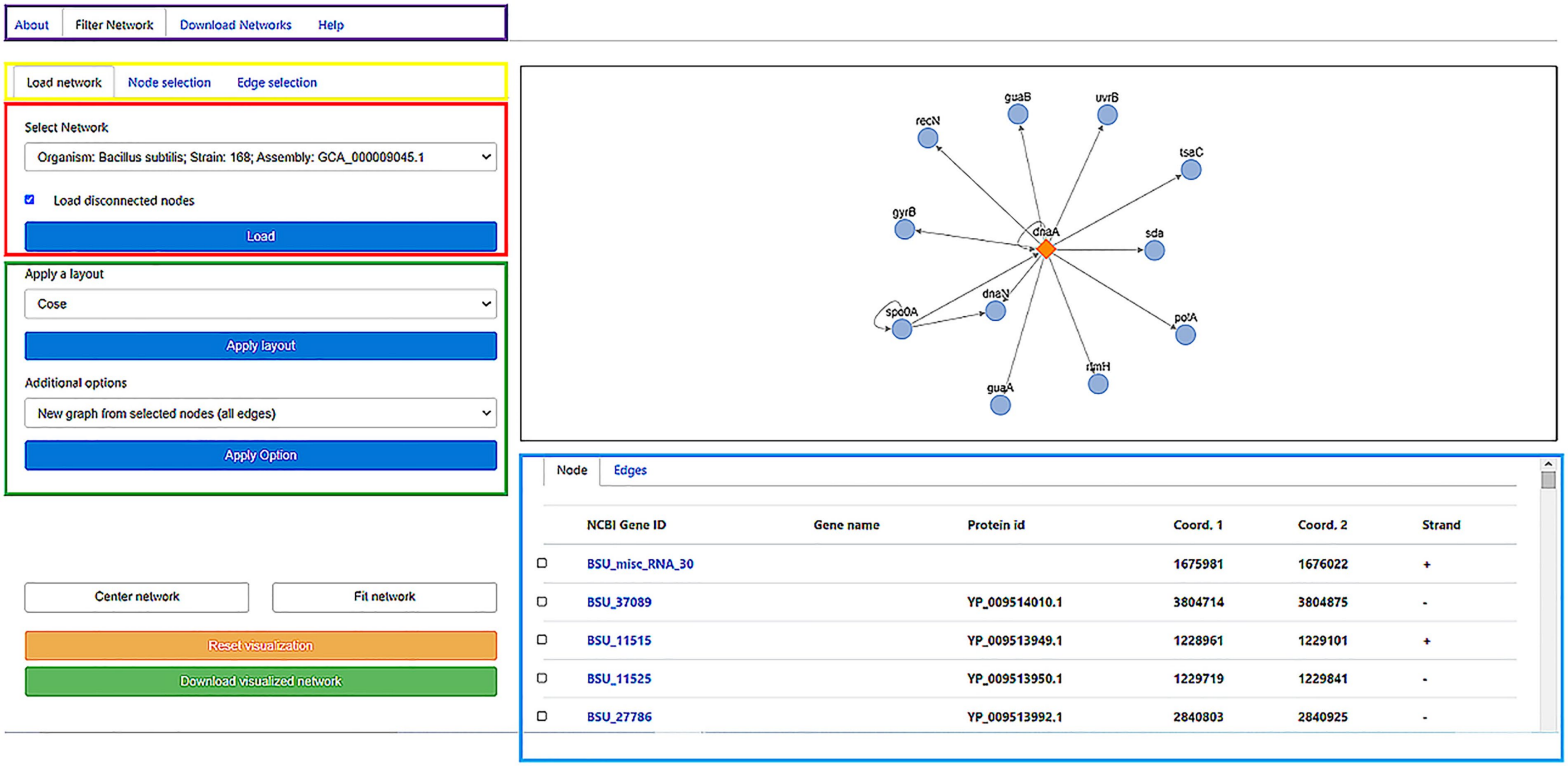
Online interface of the “regulatory networks” server storing the publicly available database. Diverse options are available for the user: a description of the system, a page to download the raw data, and the core section of the web to filter a GRN (purple box). To visualize load a network, the user can select the Gene Regulatory Network of the organism of interest in the “load a network” panel. In the Select network box, the user can *Start* selecting the name of the organism and click on the *Load* button to visualize the network on the right window (red box). This action will load the graph (black box) and node/edges properties (cyan box). Diverse layouts can be applied to visualize the network and specific nodes/edges to generate a new subgraph (green box) can be selected. As the graph visualization could be modified, the user can center/fit the network (White buttons) or reset the current visualization (Yellow button). Finally, for displayed nodes and edges, the user can download this network (Green button). In the example, in the right panel, the network is associated with the transcription factor DnaA (diamond) and their Target Genes (circles). Edges represent the transcription direction (when it is available). In the low panel, the TGs under the regulation of the TF are shown: NCBI ID, gene name, protein ID, start and end position, and strands. For more details of the web application, please refer to the Supplementary material.

The GRNs inferred in all the bacterial and archaeal genomes are available through a web server whose interface is shown in Figure 2. The GRN of user-selected organisms are shown in an embedded interactive display that through a very intuitive mouse-based interface allows the user to select subnetworks and different types of regulatory interactions. Edge and node colors can also be redefined, as well as the layout used in the network visualization, depending on their properties. Additionally, the user can display and visualize information related to Genes (name, protein ID, initial and end coordinates, and strand), and edges among nodes representing genes, including information about whether this is a new or known edge and the organism from which it was derived. Additionally, if information is available, by clicking on the node name or protein identifier, you can access the NCBI/Uniprot page related to the gene of interest.

Entire GRNs or used defined subnetworks can be downloaded in standard format for further inspection with tools such as Cytoscape, that in addition, connect our tool to the whole array of apps already available for this visualization tool. For more information and a more detailed description of both the input and output files, see the website https://regulatorynetworks.unam.mx/ or http://132.247.46.6/, help section, where an example is provided.

## Conclusion

In this work, we have expanded the GRNs for six model organisms, by considering orthologous inference and TU assignments. This inference is based on the assumption that orthologous TFs generally regulate the expression of orthologous TGs (Yu et al., 2004; Galán-Vásquez et al., 2011; Lenz et al., 2020; Soberanes-Gutiérrez et al., 2021). The inferred interactions were included in the GRN, and their topological properties were calculated. In a second step, we inferred the GRNs for 12, 879 genomes, based on TF-TG orthology relationships of six bacterial species with well-known regulatory interactions and TU assignments. We discuss some examples to show the most relevant results obtained from this inference, and topological metrics are calculated for these networks. Therefore, our approach to reconstruct regulatory networks is a valuable resource of regulatory interactions occurring within bacteria and archaea cellular domains, and it may integrate with global expression data available for these organisms in order to improve global interaction data models. From an evolutionary perspective, the dynamics to expand or modify the repertoire of cellular functions that transcription factors control involves: (a) transcriptional rewiring whereby the promoters of orthologous genes in related species differ in the presence or absence of a binding site(s) for a conserved transcription factor(s); (b) embedding horizontally acquired genes under regulation of an ancestral transcription factor; (c) restructuring of the promoters controlled by a transcription factor; and (d) modifications in the transcription factors themselves (Perez and Groisman, 2009; Pérez-Rueda et al., 2009). In this context, the inference of archaeal GRNs was based under the hypothesis that bacteria and archaea share a common ancestry in terms of their TFs, with posterior divergence (Bell and Jackson, 2001; Minezaki et al., 2005), whereas the origin of the ancestral basal transcriptional machinery cannot be ascertained, and it could have been bacterial or archaeal–eukaryal type. For instance, 53% of the total repertoire of archaeal TFs exhibit at least one homologue in bacterial genomes. In particular, archaea and clostridia share a common set of TFs classified in diverse evolutionary families (Kyrpides and Woese, 1998; Bell, 2005; Pérez-Rueda and Janga, 2010), different to the families shared with several Actinobacteria and some Gammaproteobacteria. This reinforces the notion that TFs of bacteria and archaea share a common ancestry and highlight a close relationship between the TFs from archaea and Firmicutes. In addition, bacteria and archaea share an operonic organization (Seitzer et al., 2020; Sueda et al., 2021). Thus, the experimental information concerning GRN in archaea is limited. For instance, the GRN of *Pyrococcus furiosus* shows seven regulons and 279 genes, which represent 13.5% (279 genes) of the total genes in this archaeon (Denis et al., 2018). Therefore, inferences of GRN are central to explore in detail the organisms included in this cellular domain.

Finally, we have provided readers with a website where all the networks can be analyzed and downloaded.

## Data availability statement

The original contributions presented in the study are included in the article/Supplementary material, further inquiries can be directed to the corresponding authors.

## Author contributions

All authors listed have made a substantial, direct, and intellectual contribution to the work and approved it for publication.

## Funding

This work was supported by Dirección General de Asuntos del Personal Académico-Universidad Nacional Autónoma de México (IN-209620) and CONACYT (320012) and Agencia Nacional de Investigación Científica y Desarrollo (ANID) FONDECYT 1181089 awarded to AM. Powered@NLHPC: this research was partially supported by the supercomputing infrastructure of the NLHPC (ECM-02) and the computing infrastructure of the Centro de Genómica y Bioinformática, Universidad Mayor.

## Conflict of interest

The authors declare that the research was conducted in the absence of any commercial or financial relationships that could be construed as a potential conflict of interest.

## Publisher’s note

All claims expressed in this article are solely those of the authors and do not necessarily represent those of their affiliated organizations, or those of the publisher, the editors and the reviewers. Any product that may be evaluated in this article, or claim that may be made by its manufacturer, is not guaranteed or endorsed by the publisher.

## Footnotes

^1^ https://dbtbs.hgc.jp

^2^ http://regulondb.ccg.unam.mx

^3^ www.regulome.pcyt.unam.mx

^4^ uniprot.org
