# Supplementary material for: Homology-based reconstruction of regulatory networks for bacterial and archaeal genomes
Source: Front Microbiol. 2022 Jul 19;13:923105. doi: 10.3389/fmicb.2022.923105 (PMC9344073; doi:10.3389/fmicb.2022.923105)
Supplement: Supplementary file 2 [file Data_Sheet_2.PDF]

# Homology-based reconstruction of regulatory networks for bacterial and archaeal genomes

**Luis Romero[1], Sebastian Contreras-Riquelme[2], Manuel Lira[3], Alberto J. Martin[2], Ernesto Perez-Rueda[4]**

**Affiliations**

[1]LCG-CCG, UNAM

[2]Laboratorio de Biología de Redes, Centro de Genómica y Bioinformática, Facultad Ciencias, Ingeniería y Tecnología, Universidad Mayor, Chile.

[3] Cómputo Académico. Facultad de Ciencias - UMDI-Sisal. Sede Parque Científico y Tecnológico de Yucatán. Universidad Nacional Autónoma de México.

[4]Instituto de Investigaciones en Matemáticas Aplicadas y en Sistemas, Universidad Nacional Autónoma de México, Unidad Académica Yucatán. Mérida, Yucatán. México.

*** Correspondence:**
Ernesto Perez-Rueda
ernesto.perez@iimas.unam.mx

Alberto Martin

alberto.martin@umayor.cl

## *Regulatory network web server*

This document describes our platform, how to navigate through networks, display options, and download the data.

### *Loading a network*

To display a network, you must click on the "*Filter Network*" tab.

In this tab, you will see the core of our web application, where you will be able to select the Gene Regulatory Network of your organism of interest. Start selecting the name of the organism in the Select network box. Once selected clic on the Load button and you will visualize the network on the right window

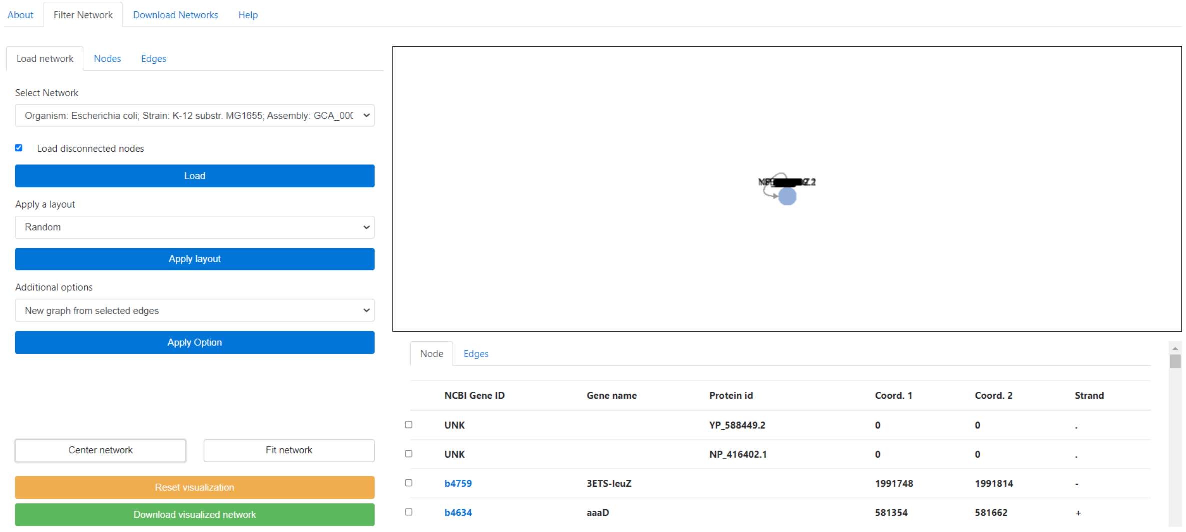


**Note:** Some genes/nodes are not connected to the other genes in the network because we do not have information about their regulation. If you want to avoid the study of these genes you need to uncheck the "Load disconnected nodes" box.

### *Getting node and edge information*

Each node in the network represents a gene, while each edge represents a regulatory process. Therefore, the "Node" panel will display information describing each gene, including the NCBI gene ID, the Gene Name (Symbol), the ID of the protein coded by the gene and its starting and end coordinates in the genome.

**Note:** be aware that it may not be possible to retrieve some information for some genes.

By looking closely to the Node table, you will be able to see that some Gene and Protein IDs colored in blue. This indicates that by clicking on them you will be redirected to their NCBI or UniProt pages.

You can also visualize the table describing the edges, just click on the "Edges" tab as shown in the image. This table shows you information such as the source and target node, the edge name if the edge is Known or derived from another organism (New) and the organism from it was derived.

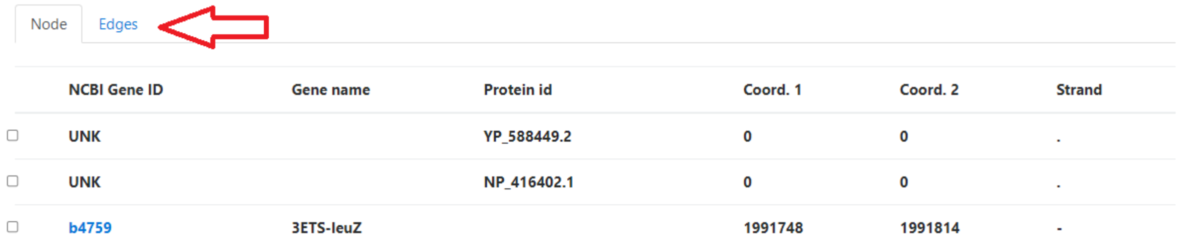

## *Selecting nodes and/or edges*

If you are interested on a certain node or edge, you can select it by cliking on the box at the left side of the row in its respective table, as shown in the next image

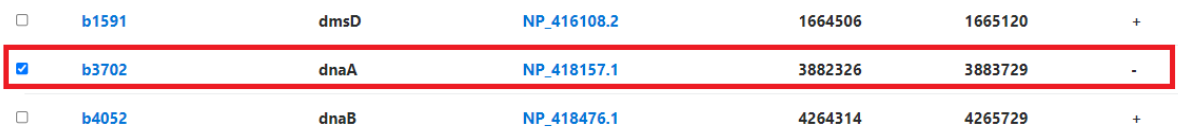


The process of manually selecting network elements can be time consuming. Therefore, to speed up this process you can go to the "Node/edge selection" tab. A easier way to perform this search is to go to the “node/edge selection” tab, There, just type a term to search, specifying the table to search. For example, to select all interactions for the *dnaA* gene, got to the edge selection tab, type "*dnaA*" and then select the property "Edge Name" and "contain". To finish, click on "Select edges" and you will see something similar to the follow image

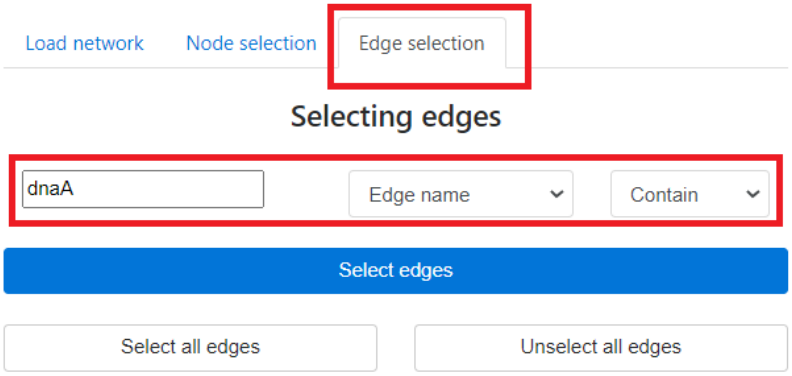


## *Selecting a subnetwork*

It is possible to create a subnetwork that contains only selected edges and nodes by clicking on the "Load network" tab. Then, click on the "additional options" tab where you will have to select edges and or nodes. You will be able to choose "New graph from selected edges" or "New graph from selected nodes", or "New graph from selected nodes and edges"

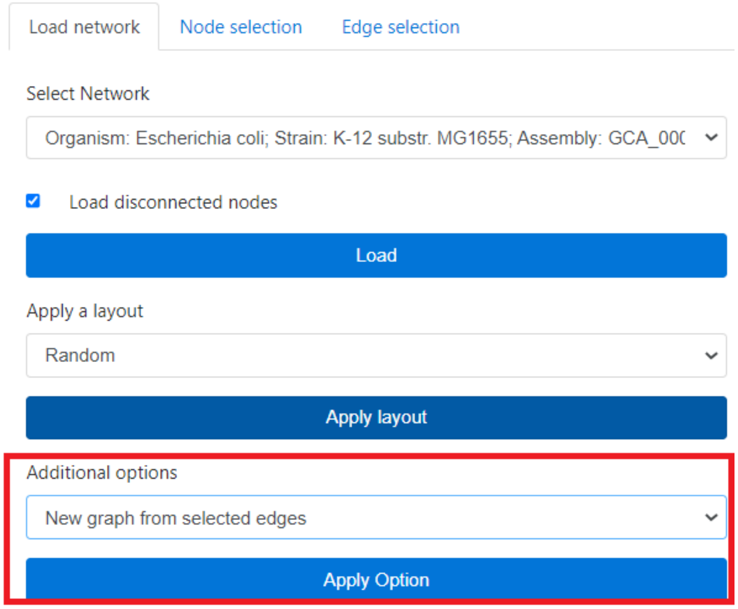


### *Applying styles*

Once you are visualizing a whole network or a subnetwork it is possible to change its layout. Please consider that it may take time to change the layout of medium size to large GRNs. Simply, select a layout style and then click on the "Apply layout" button as shown in the next image

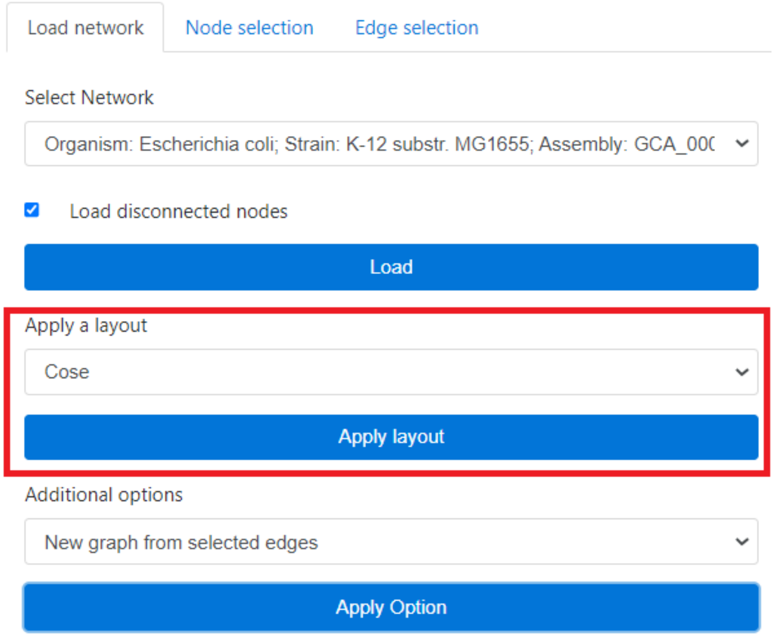


In this fashion, we will change the current layout as illustrated as follow

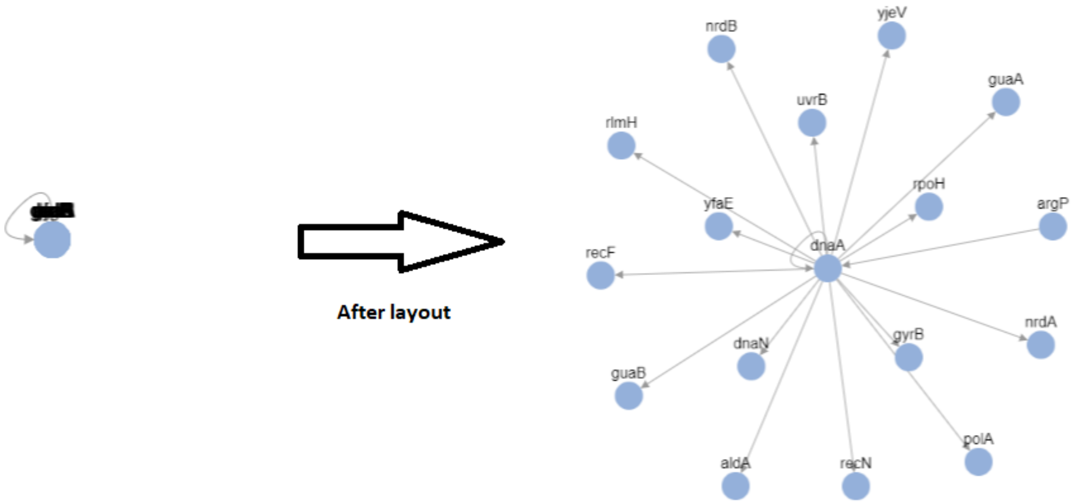


Additionally, you can also modify other aspects of the network, such as the shape and color of a node. For example, we are going to change the shape of the "*dnaA*" node to a yellow diamond and the color of the its self-loop to red. First, you must go to the "Node selection" tab, then search "dnaA" in the "Gene Name property" to then click on the "Select nodes" button.

Second, click on the "Change node color" property and choose yellow and in the "node shape" select "Diamond". After this, click on "Apply style for selected nodes". See the following images for a visual description.

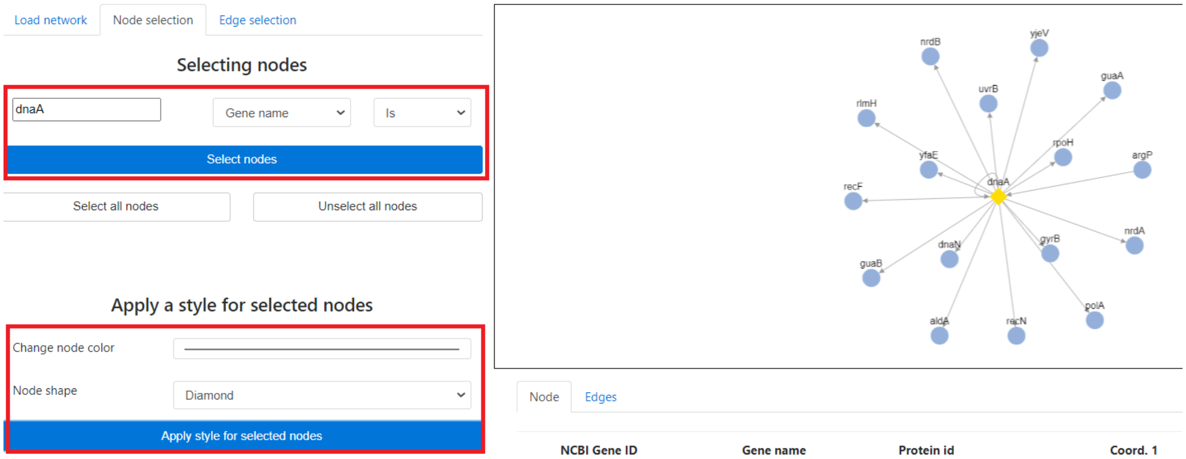


Similarly, select the self-loop edge by searching with "dnaA (interact) dnaA", and then select red as its color. Finally, just click on the “Apply style for selected edge”. All these steps and the current result are shown in the following image.

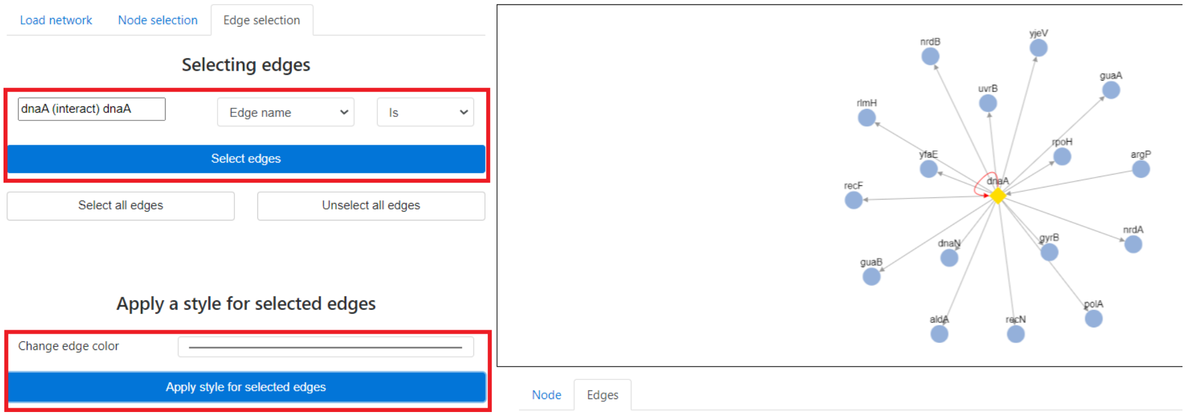


Navigating the network

To modify the size of the network, or the position of some of its nodes, just use the mouse wheel to change the network size or click on any node and then drag it to wherever you want.

The user can also re-center the network or fit it into the visualization window. Another possibility is to reset the network visualization by using the "Reset visualization" on the "Load Network" tab.

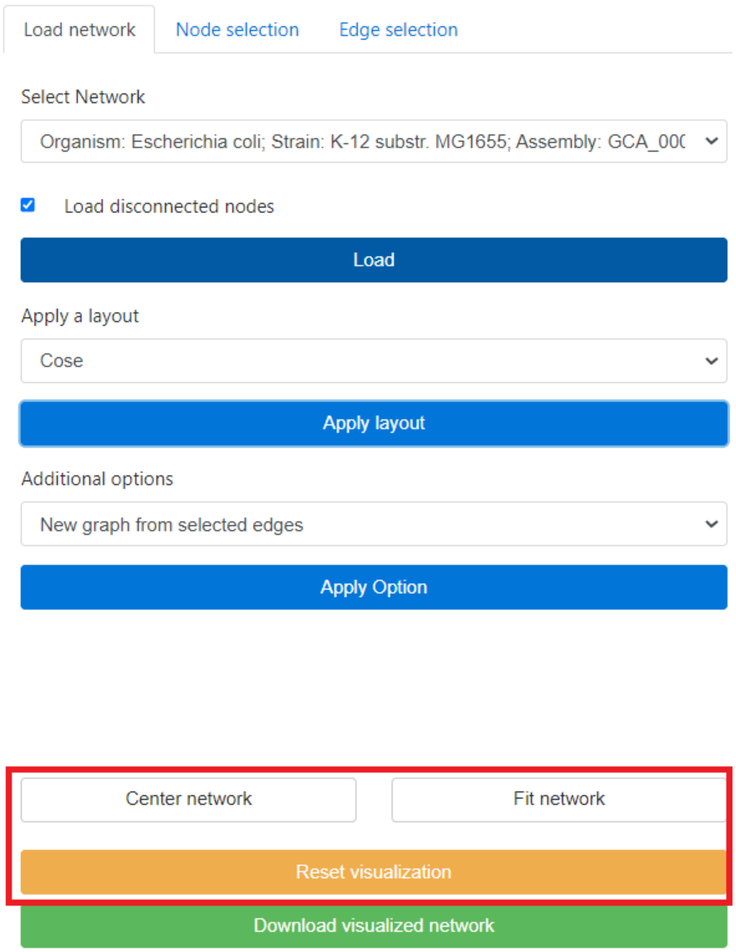


### *Downloading networks*

To download the networks, two different options are available. The first one is to download the network that is in the visualization window by clicking on the "Download visualized network" button shown in the next image.

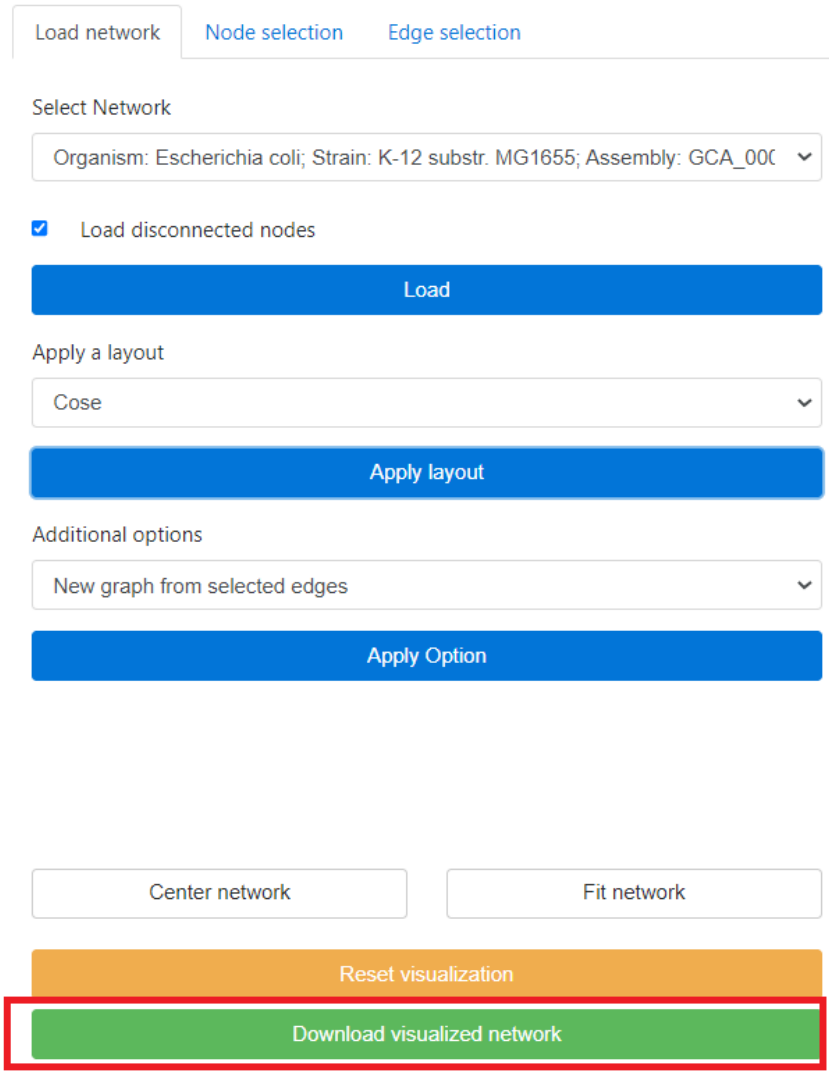


If you want to download the whole network, you have to navigate to the "Download networks" tab, where you will be able to select the network to download.

## Download networks.

In this section you can download the original networks for all organism in study.

## Data description

Each file/network is a table, where each column is separated by a tab character. Each columns represent the following features:

1. Identifier. If the edge is found in the modified network, the ID will be the same, otherwise if the edge is considered as a new one (predicted edge) will have a digit (stating with 1) and assigning status "_new"
2. Transcription factor (TF)
3. Target Gene (TG)
4. Category of the interaction: Known or predicted ("New")
5. Organism where orthologs were found for the current edge
6. ID of the homologous edge described in the modified network of the organisms mentioned in the previous point
7. Number of organisms where the current interaction exist

| Organism | Download link |
|---|---|
| Bacillus subtilis | download |
| Mycobacterium tuberculosis | download |
| Escherichia coli | download |
| Salmonella sp. | download |
| Pseudomonas aeruginosa | download |
| Staphylococcus aureus | download |
